# Regulatory Variation at Glypican-3 Underlies a Major Growth QTL in Mice

**DOI:** 10.1371/journal.pbio.0030135

**Published:** 2005-04-05

**Authors:** Fiona Oliver, Julian K Christians, Xiaojun Liu, Susan Rhind, Vinesh Verma, Claire Davison, Steve D. M Brown, Paul Denny, Peter D Keightley

**Affiliations:** **1**University of Edinburgh, Institute of Evolutionary BiologySchool of Biological Sciences, EdinburghUnited Kingdom; **2**Royal (Dick) School of Veterinary Studies, Easter Bush Veterinary CentreRoslin, MidlothianUnited Kingdom; **3**Medical Research Council Mammalian Genetics Unit, HarwellOxfordshireUnited Kingdom; University of OxfordUnited Kingdom

## Abstract

The genetic basis of variation in complex traits remains poorly understood, and few genes underlying variation have been identified. Previous work identified a quantitative trait locus (QTL) responsible for much of the response to selection on growth in mice, effecting a change in body mass of approximately 20%. By fine-mapping, we have resolved the location of this QTL to a 660-kb region containing only two genes of known function, *Gpc3* and *Gpc4,* and two other putative genes of unknown function. There are no non-synonymous polymorphisms in any of these genes, indicating that the QTL affects gene regulation. Mice carrying the high-growth QTL allele have approximately 15% lower *Gpc3* mRNA expression in kidney and liver, whereas expression differences at *Gpc4* are non-significant. Expression profiles of the two other genes within the region are inconsistent with a factor responsible for a general effect on growth. Polymorphisms in the 3′ untranslated region of *Gpc3* are strong candidates for the causal sequence variation. *Gpc3* loss-of-function mutations in humans and mice cause overgrowth and developmental abnormalities. However, no deleterious side-effects were detected in our mice, indicating that genes involved in Mendelian diseases also contribute to complex trait variation. Furthermore, these findings show that small changes in gene expression can have substantial phenotypic effects.

## Abstract 2

The genetic basis of variation in complex traits remains poorly understood. Here, a genetic variant is discovered that leads to increased body size in mice

## Introduction

Understanding the mechanisms that underlie phenotypic variation within species is crucial to addressing fundamental issues in medicine, agriculture, and evolutionary biology [1]. Identifying genes that contribute to variation in traits affected by multiple genetic and environmental factors has proven extremely difficult [2], although the molecular basis of a few quantitative trait loci (QTLs) has been elucidated [3,4,5]. Despite these successes, several general questions remain, such as whether genes involved in Mendelian disorders also contribute to complex trait variation [6], and the extent to which coding sequence versus regulatory variation is responsible for complex trait variation. In cases where there is heritable variation in gene expression, it is not clear what magnitude of difference is sufficient to contribute to phenotypic variation without substantial deleterious effects. These issues are particularly relevant to the further identification of genes responsible for complex trait variation. Numerous studies use expression microarrays to identify genes underlying trait variation [7,8,9,10], and yet such approaches will not detect coding sequence variation or subtle differences in expression.

An archetypal model for complex trait variation is body size, but with the exception of a few Mendelian mutations [11,12], no gene contributing to quantitative variation in this trait has been identified in animals. In previous work that examined lines of mice divergently selected for body size, we showed that much of the selection response is due to a large-effect QTL on chromosome (Chr) X that causes an approximately 20% difference in growth rate between homozygotes [13,14] and explains 14% of the phenotypic variance at 6 wk in an F_2_ cross between the selection lines [15]. A large X-linked effect was observed in replicate selection lines derived independently from the same base population [13], indicating that the QTL is not due to a mutation that occurred during the selection process. Rather, it is due to variation segregating within the initial population, which was derived from a cross between two inbred and one outbred strains. This scenario contrasts with the “high growth” mutation *(hg),* which arose during selection for increased growth rate in a different set of selection lines [16] and resulted from a disruption of *Socs2 (suppressor of cytokine signalling 2),* which eliminated the expression of this gene [12].

To determine the molecular basis of the X-linked QTL, we fine-mapped the QTL by progeny testing, searched for sequence polymorphisms in annotated coding regions, and examined the expression of all genes within the target region.

## Results/Discussion

### Fine-Mapping

**Figure 1:**
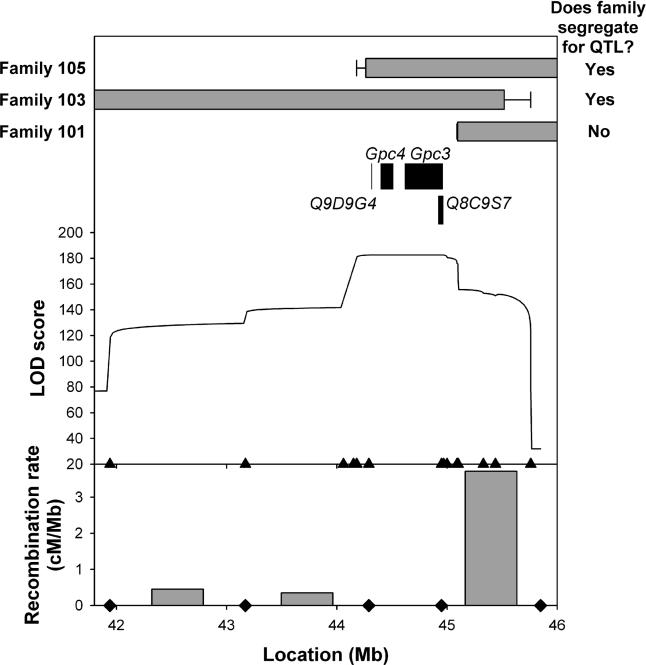
QTL Region At the top is shown the extent of Chr X segregating in three recombinant families. The horizontal grey bars indicate the regions known to segregate, while the error bars show the uncertainty in the location of recombination. Black bars indicate genes within the QTL region according to the Ensembl database [20]. Below is a LOD score plot for body mass at 6 wk in entire progeny test population (*n* = 1,909). Triangles indicate the locations of markers. At the bottom, recombination rates are shown for the intervals delimited by diamonds (the Chr X average is 0.40 cM/Mb [40]).

The QTL had previously been mapped to a region of approximately 2 cM, or 2.6 Mb [14]. By further progeny testing, we refined the location of the QTL to an approximately 660-kb region (Figure 1). The entire effect of the QTL is attributable to this region, as demonstrated by three recombinant families: families 103 and 105 segregate for the QTL region and for the phenotypic effect of the QTL, whereas family 101 does not segregate for either (Figure 1; Table 1). In both sexes, the differences in effect size between families 103 and 101 and between families 105 and 101 are significant (*p* < 0.02 in all cases), whereas the differences between families 103 and 105 are not significant (*p* > 0.2 in both cases). Thus, in contrast to previous studies that have either found QTLs to be composed of multiple QTLs (e.g., [17,18]), or have lacked the statistical power to dissect a single QTL, this large-effect QTL is caused entirely by one small chromosomal region.

**Table 1:**
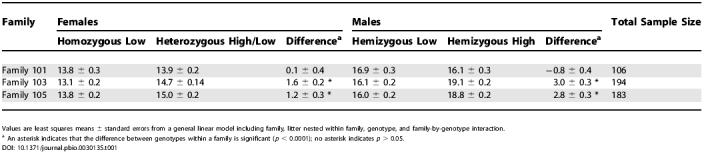
Body Mass at 6 Wk of Age in Families 103 and 105, Which Segregate for the QTL Region, and Family 101, Which Does Not

Values are least squares means ± standard errors from a general linear model including family, litter nested within family, genotype, and family-by-genotype interaction

^a^ An asterisk indicates that the difference between genotypes within a family is significant (*p* < 0.0001); no asterisk indicates *p* > 0.05

Further fine-mapping of the QTL has not been possible because the target region appears to be located in a recombination “cold spot” (Figure 1). There is substantial heterogeneity in the recombination rate within the region, roughly similar in magnitude to variation observed in humans [19], although the cold spot may be unusually wide.

### Genes within QTL Region

The QTL region contains four genes according to the Ensembl database [20], and function is known for only two of these: *Gpc3* and *Gpc4* (Figure 1). Both of these genes encode members of the glypican family of membrane-bound heparin sulphate proteoglycans that are involved in morphogenesis and growth regulation [21]. Loss-of-function mutations in *Gpc3* lead to Simpson–Golabi–Behmel syndrome in humans, a disorder with numerous phenotypic effects, including overgrowth, skeletal and renal developmental abnormalities, an increased frequency of embryonic cancers, and neonatal mortality [22,23,24]. *Gpc3* knock-out mice show similar phenotypes, including increased body mass, renal dysplasias, and increased perinatal mortality [25]. In contrast, no obvious phenotypes are seen in *Gpc4* knock-out mice [26].

DNA sequencing revealed no differences in coding sequence between the high- and low-line QTL alleles at *Gpc3, Gpc4,* or *Q8C9S7,* one of the genes of unknown function. In the other gene of unknown function, *Q9D9G4,* there was one synonymous single nucleotide polymorphism (Table S1).

### Quantitative Measurement of Expression of *Gpc3* and *Gpc4*

The lack of non-synonymous differences indicates that the effect of the QTL must be due to regulatory variation. We therefore measured mRNA transcript levels in tissues of congenic mice from litters in which the QTL segregated. Newborns were examined, since the effect of the QTL on neonatal body weight is as large as that in adults [27].

**Figure 2:**
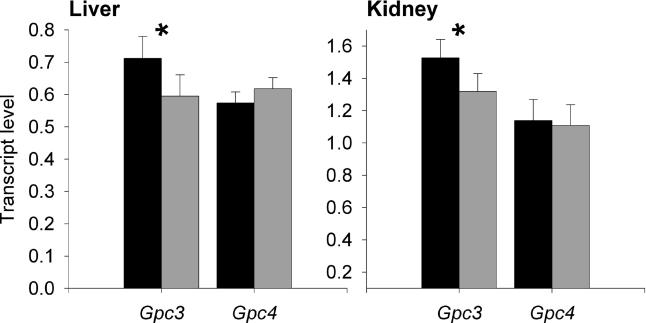
Transcript Levels of *Gpc3* and *Gpc4* (Divided By *β-actin*) Expression was measured in newborn liver and kidney in homozygous low-allele females and hemizygous low-allele males (black bars) and in heterozygous females and hemizygous high-allele males (grey bars). Data are from 23 low-allele mice and 24 high-allele males/heterozygous females, and values are least squares means (± 2× standard error); *, *p* < 0.05.

Transcript levels of *Gpc3* and *Gpc4* were examined in kidney and liver, since both genes are expressed in these tissues in mice [28], and kidney abnormalities are often observed in *Gpc3* loss-of-function mutations in humans and mice [22,23,25]. Mice with the high-line allele showed 15% lower expression of *Gpc3* in liver and kidney (*p* = 0.017 and *p* = 0.012, respectively, from a general linear model fitting effects of genotype, sex, and litter; Figure 2), whereas the differences in transcript levels for *Gpc4* were non-significant (*p* = 0.08 and *p* = 0.74, respectively), and the trends varied in direction between tissues (Figure 2; Table S2). Transcript levels of *Gpc3* and *Gpc4* were adjusted by dividing by *β-actin* levels; correcting for *β-actin* by including it as a co-variate in the model yielded qualitatively similar results. The lower level of expression of *Gpc3* in mice with the high body mass QTL allele is consistent with the overgrowth seen in *Gpc3* knock-out mice [25].

Given the phenotypic effects of the QTL (Table 1), we would expect the difference in *Gpc3* expression between hemizygous low-allele males and hemizygous high-allele males to be greater than the difference between homozygous low-allele females and heterozygous females. Although there appeared to be some indication of sex-specific differences in *Gpc3* expression in liver (Table S2), this was largely due to a marginally non-significant sex-by-genotype interaction in *β-actin* levels used to normalise *Gpc3* expression (*p* = 0.06), which generated the pattern shown in Table S2; untransformed liver *Gpc3* levels did not show a significant sex-by-genotype interaction (*p* = 0.13). The lack of significant sex-specific differences in *Gpc3* expression is likely due to low statistical power to detect interactions.

### Expression Profiling of Genes of Unknown Function

To examine whether the two genes of unknown function might contribute to the effect of the QTL, we examined their expression using a 24-tissue gene-expression panel. *Q9D9G4* (also known as *1700080O16*) was originally identified in adult male testis cDNA [20], and we observed clear expression in this tissue, as well as very low levels of expression in muscle, lung, and small intestine; no expression was detected in embryos. Others have also found much greater expression of this gene in testis than in any other tissue in a 61-tissue panel [29] and in a 55-tissue panel [30].

*Q8C9S7* (also known as *A630012P03*) was originally identified in 3-d neonate thymus cDNA [20], and while we were able to detect very low levels of expression in the thymus of 3-d-old mice, we were unable to detect its expression in any adult tissue or embryonic stage using the commercially available expression panel. *Q8C9S7* could not be found and/or there was inconsistent annotation in other expression panels [29,30]. Furthermore, this gene appears to be homologous to an annotated human pseudogene (AF003529.2) [20]. Because genes of unknown function with restricted patterns of expression did not present strong candidates for the causative factor underlying a QTL with a general effect on growth rate in both sexes, we did not pursue these two genes further.

### Expression of a Gene Downstream from *Gpc3*

To investigate the pathways through which *Gpc3* might exert its effect, we examined the expression of *Smad6 (mothers against decapentaplegic homolog 6); Gpc3* has been shown to affect BMP-7 (Bone morphogenic protein 7) signalling [31], which in turn promotes the expression of *Smad6* [32]. However, *Smad6* transcript levels did not differ significantly between genotypes in newborn liver or kidney (data not shown), suggesting that *Gpc3* exerts its effect through a different pathway. Glypican-3, the protein encoded by *Gpc3,* has been shown to bind to FGF-2 (fibroblast growth factor 2) [32,33]. Therefore, the lower *Gpc3* levels in mice with the high-line allele may lead to higher levels of unbound FGF-2 or other growth factors that may promote growth. However, insulin-like growth factors do not appear to be targets of *Gpc3* binding [33,34].

### Polymorphisms in Non-Coding DNA Adjacent to *Gpc3*

**Figure 3:**
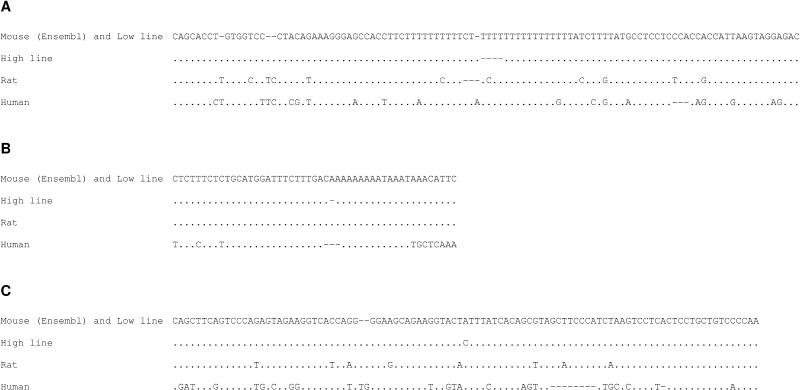
Polymorphisms between High- and Low-Line-Derived Chr X (A) 80 bp from the stop codon of *Gpc3* in the 3′ UTR. (B) 332 bp from the stop codon of *Gpc3* in the 3′ UTR. (C) 1,455 bp downstream of *Gpc3*. Sequence in common with reference mouse sequence [20] is denoted by dots.

To identify candidate polymorphisms that might be responsible for the difference in *Gpc3* transcript levels, we sequenced the 5′ and 3′ untranslated regions (UTRs), 2,876 bp upstream from the 5′ UTR of *Gpc3* (including its promoter region [35]), 1,724 bp downstream of the 3′ UTR, the first 1,048 bp of intron 1, as well as 3,377 bp of other regions of intron 1 that were identified as having high conservation with human. These are the non-coding regions near genes that show the highest levels of sequence conservation in rodents [36]. The only sequence differences between the high- and low-line-derived regions were three mononucleotide repeat polymorphisms (one in the first intron of *Gpc3* and two in the 3′ UTR), two dinucleotide repeat polymorphisms downstream from *Gpc3,* and a single nucleotide polymorphism 1,455 bp downstream of the 3′ UTR (see Table S1). This low level of polymorphism is consistent with previous findings [14] and a low frequency of microsatellite polymorphism between the lines. The 3′ UTR polymorphisms present strong candidate polymorphisms for the differential expression of *Gpc3,* since 3′ UTRs are known to play a role in mRNA stability [37,38]. For instance, the 3′ UTR of *dally,* a *Drosophila* member of the glypican family, affects the mRNA levels of this gene [39]. Furthermore, the polymorphic segments of the 3′ UTR show high conservation across mammals (Figure 3A and B). A BLAST search of a 450-bp region surrounding the downstream single nucleotide polymorphism yielded hits in the region of *Gpc3* in both human and rat, and indicated that this base pair is also conserved across these species (Figure 3C). While the 3′ UTR polymorphisms are promising candidates, it should be noted that the causative polymorphism(s) may be located further upstream or downstream than was sequenced, or in an intron (e.g., [4]).

### Pleiotropic Effects of Altered *Gpc3* Expression

Knock-out mutations of *Gpc3* generate a range of pathological phenotypes, and it might be expected that QTL-associated regulatory variation at *Gpc3* would generate milder forms of these pleiotropic effects. We therefore conducted post-mortem and histological analyses on a sample of 34 age- and sex-matched individuals. Some of the most prominent pathological conditions of *Gpc3*-deficient mice are cystic and dysplastic kidneys, imperforate vaginas leading to swelling of the perineum and fluid-filled uteri, and susceptibility to respiratory infections [25]. However, there was no evidence of cystic medullary dysplasia resembling that seen in the *Gpc3*-deficient phenotype in mice carrying the high- or low-line allele. Although a range of incidental and pathological features were recorded (Table S3), no phenotype was consistently associated with either genotype.

Since *Gpc3*-deficient mice have a reduced survival probability to weaning [25], we compared the numbers of high- and low-genotype mice surviving to weaning age in segregating litters. There is no evidence of a significant effect of genotype on numbers of high- and low-allele mice at weaning (512 and 554, respectively; χ^2^_1_ = 1.65; *p* = 0.2). For litter size, congenic females homozygous for the high-line QTL allele have somewhat higher performance than females homozygous for the low-line allele (mean ± standard error, 5.38 ± 0.22 versus 4.78 ± 0.18, respectively; *t*_198_ = 2.13; *p* = 0.03).

### Conclusions

In this study, we fine-mapped a growth QTL to a region containing only two genes of known function, found no coding sequence variation in these two genes, and demonstrated significant differences in the transcript levels of *Gpc3*. The phenotypic and expression differences between QTL genotypes are consistent with known loss-of-function mutations and knock-out phenotypes (i.e., reduced or absent *Gpc3* expression leads to increased body size). These results underscore the potential impact of relatively small changes in expression levels on phenotype.

Our results show that a gene underlying a Mendelian disease in humans can contribute to quantitative variation in mice. Unlike loss-of-function mutations, allelic variation in *Gpc3* had no pathological side-effects that we were able to detect; it affected growth rate only, and did so at all ages and in all tissues that we studied [27]. This work provides further evidence that the glypicans are involved in normal growth processes in addition to their role in Simpson–Golabi–Behmel syndrome and a variety of cancers [32].

## Materials and Methods

### 

#### Experimental mice

The inbred low line and a congenic for a high-line segment of Chr X were described previously [14]. We continued marker-assisted backcrossing to the low line to produce an interval-specific congenic strain containing a 14-cM segment of Chr X from the high line on the low-line background, with a contribution from high-line autosomes of less than 0.1%. The mice used in this study were at backcross generation 10–12. All experiments were carried out in accordance with U.K. Home Office regulations.

#### Progeny testing

Heterozygous females from the interval-specific congenic strain were crossed with low-line males, and mice recombinant between DXMit226 and DXMit68 were used for progeny testing. Recombinant males and females were crossed with low-line mice to produce families that segregated for the recombinant segment. Body weights at 6 wk of age from the progeny were recorded and flanking markers genotyped. Further genotyping using a range of microsatellite markers established the recombination breakpoints; microsatellite primer sequences are available in Table S4. PCR genotyping was carried out on DNA extracted from ear clip or tail clip samples [14].

#### Maximum likelihood analysis

The marker allelic states and phenotypes of the progeny test dataset were analysed by maximum likelihood interval mapping [14]. Briefly, each recombination event was assumed to have been replicated across litters, and the phenotypic and flanking marker data at a given chromosomal position were used to estimate a hemizygous effect in males; homozygous and heterozygous effects in females; normally distributed litter effects; and effects for litter size, parity, and sex. Likelihood ratio for the model with a QTL relative to that for the reduced model with no QTL was calculated every 0.1 cM in the region of interest, and converted to a LOD score. There were 937 males and 972 females in the dataset.

#### Post-mortem and histology analysis

A total of 34 mice, matched for genotype and sex, were sacrificed between 8 and 16 wk of age and immediately underwent a comprehensive post-mortem and histological investigation. Tissue samples were fixed in 10% phosphate-buffered formalin and processed routinely. Sections were cut at 4 μm and stained with haematoxylin and eosin. Samples of all major organ systems were examined (urinary, cardiovascular, respiratory, alimentary, endocrine, reproductive, haemolymphatic, integumentary, musculoskeletal, and central nervous systems). Standard histopathological analysis was carried out and morphologic abnormalities recorded (see Table S3).

#### DNA sequencing

Sequencing was carried out in forward and reverse directions using DYEnamic ET Terminator Cycle Sequencing Kits (Amersham Biosciences, Little Chalfont, United Kingdom) on an ABI Prism 3730 DNA Analyzer (Applied Biosystems, Foster City, California, United States) according to manufacturer's instructions. Sequencing primer sequences are shown in Table S5. *Gpc3* is a large gene with almost 340 kb of intronic sequence. We therefore sequenced only a subset of the intronic regions, focusing on regions with high sequence conservation between mouse and human to increase the likelihood of finding functional sequences. Conserved regions were identified using the “Detailed view” of ContigView at the Ensembl Web site [20] (displayed using the “Compara” menu).

#### RT-PCR

Transcript levels were examined in kidney and liver from 47 newborn mice from seven litters that were segregating for the QTL region (23 low-allele mice and 24 high-allele males or heterozygous females). Tissue samples were collected into RNAlater solution (Qiagen, Valencia, California, United States) and stored at −20 °C until required. Total RNA was isolated from tissue using Qiashredder homogenisers (Qiagen) and RNAEasy Extraction kits (Qiagen) according to manufacturer's instructions. We performed RT-PCR using One Step RT-PCR kits (Qiagen) with the addition of RNAsin RNase inhibitor (Promega, Madison, Wisconsin, United States). Reaction conditions were optimised for each gene tested and for each tissue type to ensure the PCR reactions did not reach saturation. Specifically, we determined the number of PCR cycles and starting RNA concentration such that the amount of product varied linearly with RNA concentration. RT-PCR primer sequences are provided in Table S6, and RT-PCR conditions are listed in Table S7. To check for DNA contamination, 5 μl of each RT-PCR product was run out on a 1% agarose gel.

Although no splice variants of *Gpc3* or *Gpc4* are known, we designed three primer pairs for each gene (for *Gpc3,* these spanned introns 2, 3, and 7; for *Gpc4,* these spanned introns 1, 3, and 8). For both genes, all three primer pairs yielded products of the expected size from “high” genotype RNA. Furthermore, because we sequenced the coding region using cDNA (see Table S5), we know that the entire genes are expressed for both alleles. Quantitative measurement of expression levels was performed using only one of the primer pairs per gene (see Table S6).

#### RT-PCR product quantification by DHPLC

RT-PCR products were quantified using a WAVE denaturing high-pressure liquid chromatography instrument at an oven temperature of 50 °C. We sampled 5 μl of each RT-PCR product on a DNASep column. Samples were eluted from the column using an acetonitrile gradient in a 0.1 M triethylamine acetate buffer (pH 7), at a constant flow rate of 0.9 ml min^−1^. The gradient was created by mixing eluent A (0.1 M triethylamine acetate and 0.1 M tetrasodium EDTA) and eluent B (25% acetonitrile in 0.1 M triethylamine acetate) according to the manufacturer's specifications (Transgenomic, Omaha, Nebraska, United States). Each litter of mice was measured for all three genes in one assay to eliminate variation due to differences between runs. Transcript levels of *Gpc3* and *Gpc4* were expressed relative to that of *β-actin* by dividing the amount of *Gpc3* or *Gpc4* product by that of *β-actin*. Because the RT-PCR and quantification provided only an index of transcript levels, these are in arbitrary units. All samples were analysed in triplicate and the average within-assay coefficient of variation was less than 5%.

#### Expression of genes of unknown function

The tissue distribution patterns of expression of *Q8C9S7* and *Q9D9G4* were surveyed using mouse Rapid-Scan Gene Expression panels (OriGene Technologies, Rockville, Maryland, United States; MSCB101) that included cDNA from brain, heart, kidney, spleen, thymus, liver, stomach, small intestine, muscle, lung, testis, skin, adrenal gland, pancreas, uterus, prostate gland, breast (virgin, pregnant, lactating, and involuting), and embryo (e8.5, e9.5, e12.5, and e19). The kits were used in accordance with manufacturer's instructions using an initial activation of 3 min at 94 °C; followed by 35 cycles of 30 s at 94 °C, 30 s at 55 °C, and 2 min at 72 °C; and a final extension of 5 min at 72 °C (primer sequences are provided in Table S6).

## Supporting Information

Table S1 Sequence Polymorphisms between the High-Growth Line, Low-Growth Line, and Reference Mouse Sequence [20] (41 KB DOC).

Table S2 Transcript Levels (Expressed as Ratio of *β-actin* Levels) in Newborn Mice from Litters Segregating for the QTL Values are least squares means (LSM) and standard errors (SE) from a general linear model including litter, sex, genotype, and sex-by-genotype interaction.
(40 KB DOC).

Table S3 Summary of Post-Mortem and Histological Investigations All females were homozygous high or low (i.e., not from segregating litters as in the expression and mapping studies).
(65 KB DOC).

Table S4 Microsatellite Primer Sequences Physical positions are from Ensembl Build 24.33.1 [20].
(56 KB DOC).

Table S5 Sequencing Primer Sequences Primers for *Gpc3* and *Gpc4* were designed to span introns and were used to sequence cDNA. All other primers were designed for direct sequencing of genomic DNA.
(79 KB DOC).

Table S6 RT-PCR Primer Sequences (32 KB DOC).

Table S7 RT-PCR Conditions In all cases, the RT-PCR consisted of an initial reverse transcription of 30 min at 50 °C; initial activation of 15 min at 95 °C; a variable number of cycles of 1 min at 94 °C, 1 min at 55 °C, and 1 min at 72 °C; and a final extension of 10 min at 72 °C.
(35 KB DOC).

### Accession Numbers

The LocusLink (http://www.ncbi.nlm.nih.gov/LocusLink/) accession numbers for the genes and gene products discussed in this paper are *β-actin* (LocusLink ID 11461), *BMP-7* (LocusLink ID 12162), *dally* (LocusLink ID 39013), FGF-2 (LocusLink ID 14173), *Gpc3* (LocusLink ID 14734), *Gpc4* (LocusLink ID 14735), *Smad6* (LocusLink ID 17130), and *Socs2* (LocusLink ID 216233). The Vega Gene ID (http://www.ensembl.org/) for human pseudogene AF003529.2 is OTTHUMG00000022447.

## Abbreviations

Chr: chromosome
QTL: quantitative trait locus
UTR: untranslated region
