# Supplementary material for: Regulatory Variation at Glypican-3 Underlies a Major Growth QTL in Mice
Source: PLoS Biol. 2005 Apr 5;3(5):e135. doi: 10.1371/journal.pbio.0030135 (PMC1073695; doi:10.1371/journal.pbio.0030135)
Supplement: Supplementary file 1 [file pbio.0030135.st001.doc]

Supporting Table 1. Sequence polymorphisms between the high-growth line, low-growth line, and reference mouse sequence [20].

| Location | High line  allele | Low line allele | Ensembl allele | 5' flanking sequence | Polymorphism | 3' flanking sequence | Strand shown |
| --- | --- | --- | --- | --- | --- | --- | --- |
| *Q9D9G4*  exon 4, base 81 of coding sequence | C | G | C | GGGAGACAAGAGACCTGGAGGCAGG | [C/G] | GAAGAAGAGGCCAGCACCTCAGGAG | 1 |
| 896 bp upstream of *Gpc3* 5' UTR | 22 repeats | 22 repeats | 25 repeats | GAACCACACAAAGTAAATGTGTGCA | [TG] | AATTTAGGCACTTGATCCTGATTCC | 1 |
| *Gpc3* intron 1 | 13 repeats | 14 repeats | 12 repeats | TTGGTTGGAACACACACATAGATGT | [G] | ATAGAACTCTATTTAGTAAACCTTT | 1 |
| *Gpc3* 3'UTR, 80 bp from stop codon | 13 repeats | 17 repeats | 17 repeats | AGGGAGCCACCTTCTTTTTTTTTTC | [T] | ATCTTTTATGCCTCCTCCCACCACC | -1 |
| *Gpc3* 3'UTR, 332 bp from stop codon | 8 repeats | 9 repeats | 9 repeats | CTTTCTCTGCATGGATTTCTTTGAC | [A] | TAAATAAACATTCAAATAAATGGTG | -1 |
| 29 bp downstream of *Gpc3* 3' UTR | 5 [TA] then  5 [T] | 10 repeats | 11 repeats | ATAAAGGAAGAAGTATATGACCCTT | [TA] | TTTTTAAAAAAGGACCAACACCATT | 1 |
| 556 bp downstream of *Gpc3* 3' UTR | 9 repeats | 8 repeats | 8 repeats | GCTTTTTGAGACAGTCTCTCTCTTT | [CT] | GGTCTGGAGCTCACTGAATAGGCTG | 1 |
| 1455 bp downstream of *Gpc3* 3' UTR | G | T | T | AGATGGGAAGCTACGCTGTGATAAA | [T/G] | AGTACCTTCTGCTTCCCCTGGTGAC | 1 |
