# Supplementary material for: Regulatory Variation at Glypican-3 Underlies a Major Growth QTL in Mice
Source: PLoS Biol. 2005 Apr 5;3(5):e135. doi: 10.1371/journal.pbio.0030135 (PMC1073695; doi:10.1371/journal.pbio.0030135)
Supplement: Supplementary file 2 [file pbio.0030135.st002.doc]

Supporting Table 2. Transcript levels (expressed as ratio of *β-actin* levels) in newborn mice from litters segregating for the QTL. Values are least squares means (LSM) and standard errors (SE) from a general linear model including litter, sex, genotype and sex*genotype interaction.

| Sex | Genotype | Sample size | Liver | | | | Kidney | | | |
| --- | --- | --- | --- | --- | --- | --- | --- | --- | --- | --- |
|  |  |  | *Gpc3* |  | *Gpc4* |  | *Gpc3* |  | *Gpc4* |  |
|  |  |  | LSM | SE | LSM | SE | LSM | SE | LSM | SE |
| Females | Homozygous low | 13 | 0.70 | 0.04 | 0.55 | 0.02 | 1.61 | 0.08 | 1.23 | 0.08 |
|  | Heterozygous high/low | 12 | 0.71 | 0.04 | 0.61 | 0.02 | 1.39 | 0.08 | 1.07 | 0.09 |
|  | P value^a^ |  | 0.93 |  | 0.12 |  | 0.06 |  | 0.23 |  |
| Males | Hemizygous low | 10 | 0.73 | 0.05 | 0.60 | 0.03 | 1.45 | 0.09 | 1.04 | 0.10 |
|  | Hemizygous high | 12 | 0.49 | 0.04 | 0.63 | 0.02 | 1.25 | 0.08 | 1.13 | 0.09 |
|  | P value^a^ |  | < 0.01 |  | 0.35 |  | 0.09 |  | 0.45 |  |

^a^ P values are from contrasts between genotypes within each sex.
