# Supplementary material for: Regulatory Variation at Glypican-3 Underlies a Major Growth QTL in Mice
Source: PLoS Biol. 2005 Apr 5;3(5):e135. doi: 10.1371/journal.pbio.0030135 (PMC1073695; doi:10.1371/journal.pbio.0030135)
Supplement: Supplementary file 3 [file pbio.0030135.st003.doc]

Supporting Table 3. Summary of post mortem and histological investigations. All females were homozygous high or low, i.e., not from segregating litters as in the expression and mapping studies.

| ID | Age (weeks) | Sex | Genotype | Mass (g) | Gross and Histopathological features |
| --- | --- | --- | --- | --- | --- |
| 34 | 8 | F | LOW | 14.5 | Focal dystrophic calcification in liver. Occasional degenerative and regenerative skeletal muscle fibres.  Mottled lungs. Irregular congestion. |
| 24 | 8 | F | LOW | 16.7 |  |
| 31 | 8 | F | LOW | 13.4 | Mottled lungs. Irregular congestion. |
| 26 | 8 | F | HIGH | 18.4 |  |
| 14 | 8 | F | HIGH | 18.9 |  |
| 33 | 8 | F | HIGH | 19.4 |  |
| 19 | 8 | M | LOW | 19.0 | Increased mitotic activity in seminiferous tubules |
| 22 | 8 | M | LOW | 18.5 |  |
| 25 | 8 | M | LOW | 17.3 | No sperm production |
| 9 | 8 | M | HIGH | 24.2 | Skeletal muscle - occasional degenerate and regenerative fibres. Occasional lymphoid follicles in renal cortex |
| 20 | 8 | M | HIGH | 20.1 | Increased mitotic activity in seminiferous tubules |
| 21 | 8 | M | HIGH | 21.5 | Mottled lungs. Irregular congestion. |
| 11 | 12 | F | LOW | 15.4 |  |
| 18 | 12 | F | LOW | 18.2 |  |
| 27 | 12 | F | LOW | 15.2 |  |
| 7 | 12 | F | HIGH | 21.5 |  |
| 16 | 12 | F | HIGH | 24.1 | Occasional lymphoid nodule in renal cortex. Mild enteritis and prominent lymphoid follicles |
| 29 | 12 | F | HIGH | 25.7 | Thymus neoplastic - lymphosarcoma |
| 5 | 14 | M | LOW | 21.9 | Small thyroid cyst; benign |
| 13 | 14 | M | LOW | 22.5 | Focal hepatitis and hepatic cyst. Irregular pulmonary congestion. |
| 23 | 14 | M | LOW | 23.7 | Dilated thyroid follicle. Irregular pulmonary congestion. One regenerating muscle fibre. Scattered giant cells and mitotic cells in seminiferous tubules |
| 30 | 14 | M | LOW | 22.6 |  |
| 3 | 14 | M | HIGH | 30.0 | Mottled lungs. Irregular congestion. |
| 17 | 14 | M | HIGH | 29.7 | Focal sebaceous hyperplasia |
| 32 | 14 | M | HIGH | 24.7 |  |
| 28 | 14 | M | HIGH | 29.7 | Scattered degenerate seminiferous tubules |
| 2 | 16 | F | LOW | 18.3 | Small focus of chronic active hepatitis. Neutrophils in uterine lamina propria |
| 4 | 16 | F | LOW | 19.9 |  |
| 8 | 16 | F | LOW | 16.8 |  |
| 12 | 16 | F | LOW | 19.5 | Irregular pulmonary congestion. Focal accumulations of neutrophils in uterine lamina propria |
| 1 | 16 | F | HIGH | 22.8 |  |
| 6 | 16 | F | HIGH | 24.8 | Multifocal lymphocytic interstitial nephritis (mild) |
| 10 | 16 | F | HIGH | 24.9 | Focal lymphoid aggregates in kidney |
| 15 | 16 | F | HIGH | 18.5 | Uterus distended with eosinophilic fibrillary debris and degenerate inflammatory cells |
