# Supplementary material for: Regulatory Variation at Glypican-3 Underlies a Major Growth QTL in Mice
Source: PLoS Biol. 2005 Apr 5;3(5):e135. doi: 10.1371/journal.pbio.0030135 (PMC1073695; doi:10.1371/journal.pbio.0030135)
Supplement: Supplementary file 4 [file pbio.0030135.st004.doc]

Supporting Table 4. Microsatellite primer sequences. Physical positions are from Ensembl Build 24.33.1 [20].

| Position (cM) | Position (Mb) | Primer pair name | Left sequence | Right Sequence | Approx. size (bp) |
| --- | --- | --- | --- | --- | --- |
| 0 | 39636328 | DXMit226 | CTACTCCCTGGGACAAGCAC | CACAAAGGTGAGTAGAAGATGCA | 124 |
| 0.658 | 41935562 | DXIcp13 | GACTTAGGGATGTTGTGCAT | TTGTCCCTAGGATCAAATTC | 182 |
| 1.217 | 43168194 | DXIcp20 | TTCAACTACCAAAGGGCACA | TGAGGACACAACACACACATT | 275 |
| 1.405 | 44062558 | DXIcp30 | TTTTCTTTATGTCTTTGTTTTG | ACGTACATCTTCACTTATGG | 427 |
| 1.405 | 44147989 | DXIcp35 | GGCAGCTGTTAGAAATGGTT | AAAACCTCTCAGTAAAAGTGAAAA | 300 |
| 1.405 | 44175340 | DXIcp36 | GGCTTTCGATTTTCTTCCTT | AACCAAACATTTTCAAGACCA | 331 |
| 1.611 | 44291635 | DXIcp31 | GCACAACATTGATTTGGTAGA | GGAGAGCAGAATGGAACCT | 240 |
| 1.611 | 44949790 | DXIcp49 | GAGCAAAATGCCACGATATT | TACACATGGAGAGGGGAAGA | 209 |
| 1.704 | 44973023 | DXIcp34 | TATAGGCCACCCAAGCAGT | TCAATCCTCTCTCCCTCTCA | 268 |
| 1.843 | 44996671 | DXIcp12 | GCAAGTTCCAGGACAGTCT | TGTTCTGTGTTGAGCCTAGA | 199 |
| 2.169 | 45092842 | DXIcp16 | GAAGGGATCACATGCTTTTC | AGGGCATTTTAACACACAAAG | 212 |
| 2.260 | 45095530 | mtr20 | TACCAAGGTGGGGGTAAAGG | TGGAGTGGGAAAAGAACACA | 194 |
| 2.624 | 45329989 | DXIcp15 | ACAACATGAGCCCAGAGAAA | CACCCTCATTCCCATACTGA | 195 |
| 2.985 | 45438513 | DXIcp22 | CCAAGAAATGTGTGAATGCTT | GTACCCCGCTCCATTCTATC | 235 |
| 4.901 | 45758525 | DXIcp51 | CTCCCCAAAATCTAAACTCAAG | TGCCACAGTTCTGTTTTCTTC | 199 |
| 4.918 | 45762708 | DXIcp29 | CCTCCCCCAATGATTTTTAT | GCCCCACTAAAGAGTTTGTT | 242 |
| 4.936 | 45843712 | DXIcp23 | AAGTGCAGATGTCGCTCACT | GAGAGCAGAGACAGGGATGA | 236 |
| 4.956 | 45847566 | DXMit68 | TCCTTTGGCCTCCTGCATAT | TGTTCTTACAATGAGCCTCATAGG | 128 |
