# Supplementary material for: Regulatory Variation at Glypican-3 Underlies a Major Growth QTL in Mice
Source: PLoS Biol. 2005 Apr 5;3(5):e135. doi: 10.1371/journal.pbio.0030135 (PMC1073695; doi:10.1371/journal.pbio.0030135)
Supplement: Supplementary file 5 [file pbio.0030135.st005.doc]

Supporting Table 5. Sequencing primer sequences. Primers for *Gpc3* and *Gpc4* were designed to span introns and were used to sequence cDNA. All other primers were designed for direct sequencing of genomic DNA.

| Region | Primer pair name | Forward primer | Reverse primer | Product size (bp) |
| --- | --- | --- | --- | --- |
| *Gpc3* | Gpc3-1 | GCTCCTCTCTTGCTCTGTC | CTGCCAAATACTTTCAGGTC | 733 |
| *Gpc3* | Gpc3-2 | GCCTCTTTCCAGTCATCTAC | TTCTGGCTGTATCTCTCCAC | 787 |
| *Gpc3* | Gpc3-3 | GAAAATGATACCCTGTGCTG | TGGCTCCCTTTCTGTAGG | 558 |
| *Gpc4* | Gpc4-1 | GGTCTCATTCTGTCCCCTAA | AAAATGGTACTGGGAGTTCA | 597 |
| *Gpc4* | Gpc4-2 | ATGTGAACCTGGAAGAAATG | GCTCTGGATGATAAGGTCTG | 654 |
| *Gpc4* | Gpc4-3 | AAGGTTTTCCAAGGCTGT | CATTGTTGTCCATTCATTT | 786 |
| *Gpc3* upstream | Gpc3up0 | CTGGAAGAAAGAACGGACCT | CCACCTTGGTAAACCCAATC | 476 |
| *Gpc3* upstream | Gpc3up1 | TCAGCAAGTCTGGCAGTAG | TCCCAGAGCCAGTCAGAG | 476 |
| *Gpc3* upstream | Gpc3up2 | CAATCCTGCCCTGACACTC | TGGAACGTGACTATTTGTGG | 481 |
| *Gpc3* upstream | Gpc3up23 | AGAGCCCACACAAGGTTTC | ATTGAATGAGACCCAGCAAG | 223 |
| Gpc3 upstream | Gpc3up3 | GGTTTCTTGGGAACCCTTT | AGGGCTCTACTCCAGGTATG | 414 |
| *Gpc3* upstream | Gpc3up4 | TAAAGCAAAGTGTAGCTGGTG | TAAATTGCTCAGTTGCCTTC | 517 |
| Gpc3 upstream | Gpc3up5 | GGGGCTATGAAGAAAGAGAAA | GGGGTGACTAAGCCCTCTG | 825 |
| *Gpc3* upstream | Gpc3up56 | aactaaggcaggagggagag | TATCAAACTCACCAGCCAAA | 537 |
| Gpc3 upstream | Gpc3up6 | GGACAGAGGGCTTAGTCACC | CCTCATATTTTGCTGAACTGGA | 607 |
| *Gpc3* downstream | Gpc3down2 | CAAACCTCAAATTCAGAAGCA | CTGTACGTGGCTTTTCTACCA | 475 |
| Gpc3 downstream | Gpc3down3 | ATTTCCCAGAATCAAAGAGAA | CTAAGCCGCAGCCTCAGT | 441 |
| *Gpc3* downstream | Gpc3down4 | CAGCTCCATGAGGGTAGTGA | CACACGAATTTCAAAGAGAAAA | 396 |
| *Gpc3* downstream | Gpc3down5 | AGAGCAAGAGAGAGCTGAGAGA | CCTTAGGTTGGATTGTCCTG | 548 |
| *Gpc3* downstream | Gpc3down6 | AAAGAGAGGTCATGGTGGAAC | TCTCTGCATGGATTTCTTTGA | 392 |
| *Gpc3* downstream | Gpc3down7 | GAAGGCCAGATAATAAGAGCAA | GGAAATCAGAAGGACAACGA | 578 |
| *Gpc3* downstream | Gpc3down8 | TCCAACAGTCAAATGTAAGAAA | CCATCTATGTGGCGTGCT | 315 |
| *Gpc3* intron 1 | Gpc3in1 | CTCTCCATCTTAGGGGAGTTG | TCCAATCGCATGTTTCATTT | 549 |
| *Gpc3* intron 1 | Gpc3in2 | CCCCTTCTTTAAGCACCATT | CGTTCACTGGCAGGAGGT | 475 |
| *Gpc3* intron 1 | Gpc3in3 | GAGATCAGAGTAAAAGGAAGCA | CCTTATGGGCTGGTAGATTGT | 425 |
| *Gpc3* intron 1 | Gpc3in4 | CTCAAGTGTCTGCCTGCTTT | ATTCCTTTGCACTTTGCTTG | 651 |
| *Gpc3* intron 1 | Gpc3in6 | AAGCTGGGCAGTAATGAAAG | ACCATTTGGGTTTCTTTTGG | 395 |
| *Gpc3* intron 1 | Gpc3in7 | TGGGATCTGAAGACATGGAG | CATGATATAGGCATTGCTTTC | 411 |
| *Gpc3* intron 1 | Gpc3in9 | GGAAGGAGAGCCTGGAAG | CCAAATTCATCTGCCTTGAC | 358 |
| *Gpc3* intron 1 | Gpc3in10 | TGCCCCTACAAAATGATCTG | GACTGTGACTCGGGAATTTATC | 469 |
| *Gpc3* intron 1 | Gpc3in11 | GGGACACACGGTGGATAAA | CCGTTCTTTCTTCCAGAGAC | 505 |
| *Gpc3* intron 1 | Gpc3in12 | AATCCCAATGCCTCCTTCT | TAGATGCAGTGTGTGTGCTG | 487 |
| *Gpc3* intron 1 | Gpc3in13 | TGCGGCCTCTATTATATCTTTC | CCTTTCTCCAACACTTTCTCC | 495 |
| *Q9D9G4* | Q9Dex1 | gcctctgcttcaccatctac | tggactatcctatgctaaaacc | 313 |
| *Q9D9G4* | Q9Dex2 | agtctggaagaggtgtgtgg | gtcttgacatccctgctttt | 386 |
| *Q9D9G4* | Q9Dex3 | cctgcttctggtctgtgag | cctgtgcattcctcctctt | 393 |
| *Q9D9G4* | Q9Dex41 | cacatgctggaactttgtcc | CTTTGGTACGCCTCGAAAAC | 686 |
| *Q9D9G4* | Q9Dex42 | CCGTGTCCACTCCTACTCTC | atgccatgttctttcccatt | 832 |
| *Q8C9S7* | Q8Cex1 | gcaaaatacagggagtctgg | cctctaagctggaagtggtg | 694 |
| *Q8C9S7* | Q8Cex2 | gatcacaaagatcggaaagg | atgtgtgagttgaggggttc | 398 |
| *Q8C9S7* | Q8Cex3 | tgggatctgaagacatggag | tgctttcccaaggaagtaaa | 397 |
| *Q8C9S7* | Q8Cex4 | acgctcatctgcacaaact | aggacatggggagtcaaaa | 400 |
| *Q8C9S7* | Q8Cex51 | caggaaggaaggaaactca | CAGCCATGCTATCTAAACACTT | 500 |
| *Q8C9S7* | Q8Cex512 | ATTCGAGACAAGCTCCCTTC | CATGGAACCAGACTCTCCTC | 364 |
| *Q8C9S7* | Q8Cex52 | AATCATGGCTCTGTGCTCTC | CCTCATTGCCCCTCAGTT | 645 |
| *Q8C9S7* | Q8Cex523 | GCTGACAAACTCATCCTATCTC | GCAGCAAGCAGCCTAACA | 249 |
| *Q8C9S7* | Q8Cex53 | AAGTATTTCAACAGGGATCAA | cctatgaagacacaaaatgct | 836 |
| *Q8C9S7* | Q8Cex54 | GCACACTGATCCCAATTCTC | cctatgaagacacaaaatgct | 541 |
