# Supplementary material for: Regulatory Variation at Glypican-3 Underlies a Major Growth QTL in Mice
Source: PLoS Biol. 2005 Apr 5;3(5):e135. doi: 10.1371/journal.pbio.0030135 (PMC1073695; doi:10.1371/journal.pbio.0030135)
Supplement: Supplementary file 6 [file pbio.0030135.st006.doc]

Supporting Table 6. RT-PCR primer sequences.

| Gene | Forward primer | Reverse primer | Spanning intron number |
| --- | --- | --- | --- |
| *Gpc3* | GATCTATATTGGCGTTGCTG | GTGGTCATGCAAGGCTGTA | 3 |
| *Gpc4* | GAATGGTCAGTGGCGTTGTA | CCAGAAGTCCAGGTTGACAC | 8 |
| *β-actin* | TCTGGCACCACACCTTCTAC | AGGATCTTCATGAGGTAGTCTG | 3 |
| *Q8C9S7* | CGGTGCCTATTTATGAAGGA | TGAGATTCTTCCCACCTCTG | 1 |
| *Q9D9G4* | TCCCTTTCAGAATGAGTGCT | CGCTCCCCAAACAGAAGA | 2 and 3 |
| *Smad6* | ACGCAGTGGAGCTGAAAC | CAGTGATGAGGGAGTTGGTG | 1 and 2 |
