# Supplementary material for: Regulatory Variation at Glypican-3 Underlies a Major Growth QTL in Mice
Source: PLoS Biol. 2005 Apr 5;3(5):e135. doi: 10.1371/journal.pbio.0030135 (PMC1073695; doi:10.1371/journal.pbio.0030135)
Supplement: Supplementary file 7 [file pbio.0030135.st007.doc]

Supporting Table 7. RT-PCR conditions. In all cases, the RT-PCR consisted of an initial reverse transcription of 30 minutes at 50°C, initial activation of 15 minutes at 95°C followed by a variable number of cycles of 1 minute at 94°C, 1 minute at 55°C and 1 minute at 72°C, then a final extension of 10 minutes at 72°C.

| Tissue | Gene | Total RNA per reaction (ng) | Number of cycles |
| --- | --- | --- | --- |
| Liver | *Gpc3* | 100 | 22 |
| Liver | *Gpc4* | 100 | 25 |
| Liver | *β-actin* | 50 | 20 |
| Liver | *Smad6* | 25 | 32 |
| Kidney | *Gpc3* | 20 | 25 |
| Kidney | *Gpc4* | 20 | 25 |
| Kidney | *β-actin* | 20 | 25 |
| Kidney | *Smad6* | 25 | 30 |
